# Mechanism and therapeutic effect of umbilical cord mesenchymal stem cells in inflammatory bowel disease

**DOI:** 10.1038/s41598-019-54194-y

**Published:** 2019-11-27

**Authors:** Xing-hua Pan, Qing-qing Li, Xiang-qing Zhu, Zi-an Li, Xue-min Cai, Rong-qing Pang, Guang-ping Ruan

**Affiliations:** 1 Kunming Key Laboratory of Stem Cell and Regenerative Medicine, 920th Hospital of the PLA Joint Logistics Support Force, Kunming, Yunnan Province 650032 China; 2 Stem Cells and Immune Cells Biomedical Techniques Integrated Engineering Laboratory of State and Regions, Kunming, Yunnan Province China; 3 Cell Therapy Technology Transfer Medical Key Laboratory of Yunnan Province, Kunming, Yunnan Province China

**Keywords:** Mechanisms of disease, Gastroenterology

## Abstract

Inflammatory bowel disease (IBD) is a persistent and chronic disease that is characterized by destructive gastrointestinal (GI) inflammation. Researchers are trying to identify and develop new and more effective treatments with no side effects. Acute and chronic mouse models of IBD were established using dextran sulfate sodium (DSS) solution. To evaluate the efficacy and mechanism, umbilical cord mesenchymal stem cells (UCMSCs) were obtained from Kunming (KM) mice and humans. In the chronic IBD study, the survival rates of the normal control, model, mouse UCMSC (mUCMSC) and human UCMSC (hUCMSC) groups were 100%, 40%, 86.7%, and 100%, respectively. The histopathological scores of the normal control, intraperitoneal injection, intravenous treatment, and model groups were 0.5 ± 0.30, 5.9 ± 1.10, 8.7 ± 1.39, and 8.8 ± 1.33 (p = 0.021). UCMSCs promoted the expression of the intestinal tight junction protein occludin, downregulated the protein expression of the autophagy marker LC3A/B in colon tissue, and upregulated the expression of VEGF-A and VEGFR-1 at the injured site. This study provides an experimental model for elucidating the therapeutic effects of UCMSCs in IBD. We provide a theoretical basis and method for the clinical treatment of IBD using UCMSCs.

## Introduction

Inflammatory bowel disease (IBD) is a persistent and chronic disease that is characterized by destructive gastrointestinal (GI) inflammation^1^. The main disease types that are associated with IBD include ulcerative colitis (UC) and Crohn’s disease (CD). UC is more common in people who are 20 to 40 years old, and the age of CD is usually 15–30 years. There is no significant difference in the incidence of IBD between men and women, and these diseases can present at any age. Currently, IBD is generally considered to be caused by interactions among environmental, genetic, infectious and immune factors. Researchers are trying to identify and develop new and more effective treatments to avoid the side effects of long-term medicine use.

A large number of studies have shown that mesenchymal stem cells (MSCs) have immune regulation and tissue repair functions^2–5^. MSCs have achieved certain therapeutic effects in preclinical research and clinical trials in IBD animal models. MSCs can colonize the intestinal mucosa, control inflammation and repair damaged tissues^6,7^. After intravenous (IV) injection, MSCs can home to the injury site on the intestinal wall and improve local microcirculation to accelerate tissue repair^8^. E Gonzalez-Rey *et al*. hypothesized that MSCs treat IBD by downregulating Th1-driven inflammatory responses, lowering the levels of various inflammatory molecules and chemokines. Moreover, MSCs upregulate the secretion of the inhibitory factor IL-10 by acting on macrophages while inhibiting Th1 cell activation, which promotes the production of IL-10-secreting T cells^9^. Studies have found that MSCs do not have to colonize target tissues to achieve therapeutic benefits; tumor necrosis factor–induced protein 6 (TSG6) secreted by T- and B-lymphocytes and macrophages accumulated in the peritoneum is sufficient to relieve enteritis^10^. Fei Mao *et al*. concluded that umbilical cord MSCs (UCMSCs) attenuate dextran sulfate sodium (DSS)-induced IBD by regulating 15-lipoxygenase-1 (15-LOX-1) expression in macrophages^11^. MSCs play a therapeutic role by inducing the generation of a class of macrophages with powerful immunoregulatory functions^12,13^. Some people also believe that MSCs mainly participate in tissue repair through paracrine functions, rather than through so-called lateral differentiation or cell fusion^14^. The published literature indicates that MSCs do not survive *in vivo* for a long time, with a lifetime generally ranging from a few days to tens of days^15^. The fate and metabolism of MSCs in the body and their long-term side effects are still unclear.

MSCs have also shown significant therapeutic effects in clinical trials. MSCs are safe and effective in the treatment of CD in patients with an intractable intestinal fistula. Rachele Ciccocioppo *et al*. extracted the patient’s own bone marrow MSCs (BM-MSCs), expanded them to a certain number *in vitro*, and then locally injected these cells into the lesions of the same patient. Of the ten patients who underwent MSC treatment, 7 achieved complete closure of the fistula. Although the fistula was not completely closed in the other three patients, the CD activity index (CDAI) and perianal disease activity index (DAI) were significantly improved. No adverse reactions were observed during the follow-up period^16^. The number of regulatory T cells increased in the blood and mucous membrane samples from the treated patients. Other studies have confirmed that allogeneic MSCs expanded *in vitro* are safe and effective in the treatment of CD-complicated perianal fistula lesions^17^. In China, UCMSCs were intravenously injected into patients with CD, and the CDAI, Harvey-Bradshaw index (HBI) and corticosteroid doses were evaluated. After one year of follow-up, the curative effect was obvious, although the treatment caused mild side effects, such as fever^18^.

In many animal experiments, the main treatment routes of MSCs are IV and intraperitoneal (IP) injections, followed by local lesion injection. Several treatments have proven effective^19–21^. There are few reports on the treatment of IBD with UCMSCs of heterogeneous origin. To further clarify the optimal UCMSC injection route and source, the following research was carried out.

This study aimed to prepare and identify human and murine UCMSCs (hUCMSCs and mUCMSCs, respectively). Animal models were used to evaluate the efficacy of different hUCMSC injection routes in an acute IBD model and of UCMSCs from different species in a chronic IBD model and to explore the related mechanisms. This study found that UCMSCs promoted the expression of the intestinal tight junction protein occludin, downregulated the protein expression of the autophagy marker LC3A/B in colon tissue, and upregulated the expression of VEGF-A and VEGFR-1 at the injured site.

## Materials and Methods

### Preparation of UCMSCs

1. Kunming (KM) mUCMSCs: Three pregnant KM mice were sacrificed by cervical dislocation, a laparotomy was conducted, the umbilical cords were removed, and the capsules and blood were removed. The umbilical cords were cut into pieces smaller than 1 mm^3^ and inoculated in the bottom of T25 culture flasks, which were placed in a 5% CO_2_ incubator. When the UCMSCs reached 80%-90% confluence, they were passaged at a 1:3 ratio. Experimental protocols were approved by the Experimental Animal Ethics Committee of the 920th Hospital of the PLA Joint Logistics Support Force. All methods were carried out in accordance with relevant guidelines and regulations.
2. hUCMSCs: P2 cells supplied by the Stem Cells and Immune Cells Biomedical Techniques Integrated Engineering Laboratory of State and Regions were passaged at a 1:3 ratio. The rate of positive expression of UCMSC antigens on human and mouse P3 UCMSCs was analyzed by flow cytometry, and the osteogenic, adipogenic and chondrogenic differentiation abilities of UCMSCs were analyzed. P3 cells were diluted to 3 × 10^6^ cells/ml in cryopreservation solution and stored in liquid nitrogen for later use. For experiments, UCMSCs were diluted to 5 × 10^6^ cells/ml and 1 × 10^7^ cells/ml in physiological saline. All experiments were performed in accordance with relevant guidelines and regulations. The use of human umbilical cord mesenchymal stem cells was approved by the Experimental Animal Ethics Committee of the 920th Hospital of the PLA Joint Logistics Support Force.

### Establishment of an IBD animal model

1. Acute model: Healthy male C57BL/6 mice (age: 7–8 w; body weight: 20–22 g) had free access to 3% DSS aqueous drinking water for seven days, and the mice were sacrificed.
2. Chronic model: The mice had free access to 3% DSS in drinking water for 5 days. After 10 days, the mice underwent 5 cycles of drinking DSS solution for 4 days, with a three-day interval between cycles. The mice were sacrificed, and relevant samples were collected. During the modeling period, body weight, fecal morphology, blood in the stool and mortality were monitored daily. After the model was established, HE staining of colon tissue was performed to observe the histological changes and to obtain a histopathological score.

### UCMSC transplantation for the treatment of mouse IBD

1. Different methods of injecting hUCMSCs into the acute IBD model: Thirty-six mice with acute IBD were established according to the above method. These mice were randomly divided into a model group, an IP UCMSC injection group, an IV UCMSC injection group, with 12 mice in each group; 9 additional mice formed the normal control group. The IV and IP UCMSC injection groups were injected with 100 µl of a UCMSC suspension (5 × 10^6^ cells/ml) on the first, third and fifth days after initiating the model generation. The model group was injected with the same volume of normal saline. The mice were sacrificed on the seventh day after the start of the experiment.
2. UCMSC treatment of chronic IBD: Forty-five healthy male C57BL/6 mice were used to prepare chronic IBD models according to the above method. Then, the mice were randomly divided into a model group, a mUCMSC group, and a hUCMSC group, with 15 mice in each group; 9 additional mice were placed in the normal control group. The mice in the mUCMSC and hUCMSC treatment groups were injected with mUCMSCs or hUCMSCs (1 × 10^7^ cells/ml; 100 µl) into the abdominal cavity. The mice in the model group received an IP injection of the same volume of normal saline. Mice were sacrificed on the 50th day after the start of modeling.
3. Observation indicators: ① mouse colon tissue structure; ② serum IL-6 and TNF-α concentrations and inflammatory cell immunohistochemical staining; ③ colon tissue staining for goblet cells; ④ collagen staining in colon tissue; ⑤ daily mental state and body weight of the mice; ⑥ fecal morphology; ⑦ blood in the stool; and ⑧ relative claudin-1, occludin, and IL-6 gene expression and VEGF-A, VEGFR-1, and occludin protein expression in colon tissue.

### Analysis of the mechanism of action of UCMSCs on IBD

The protein expression of the autophagy marker LC3A/B, the tight junction factor occludin, and the vascular endothelial growth factors VEGF-A and VEGFR-1 in colon tissue was detected by Western blotting. The expression of the tight junction proteins claudin-1 and occludin, inflammatory factors and IL-6 was analyzed by qPCR. Immunohistochemistry analysis of GFP-labeled UCMSCs was used to observe the distribution of UCMSCs *in vivo*.

### Statistical analysis

All statistical data were analyzed using GraphPad Prism 5 and SPSS 22. Two independent sample means were compared by the t test, and the means of measurement data were analyzed by one-way ANOVA. P < 0.05 indicated statistical significance. Data are expressed as the mean ± standard deviation.

### Ethics approval and consent to participate

Experimental protocols were approved by the Experimental Animal Ethics Committee of 920th Hospital of the PLA Joint Logistics Support Force.

## Results

### Identification of UCMSCs

**Figure 1:**
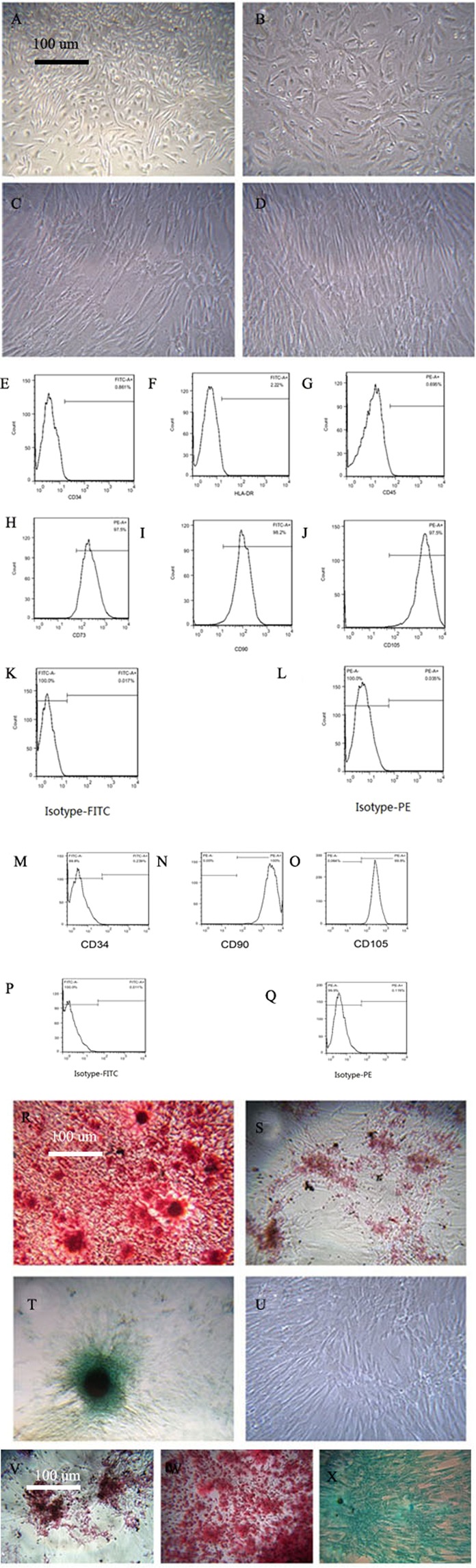
Cultivation of human and murine UCMSCs. (**A**,**B**) are P0 and P1 mUCMSCs, respectively; (**C**,**D**) are P2 and P3 hUCMSCs, respectively. The cells were spindle-shaped, and hUCMSCs were slightly larger than mUCMSCs. Flow cytometry detection of hUCMSCs. The rates of hUCMSCs positive for CD73, CD90 and CD105 were as high as 97.5%, 98.2% and 97.5%, respectively, while those for HLA-DR, CD34 and CD45 were only 2.22%, 0.861% and 0.695%, respectively. (**K**,**L**) Isotype control. Flow cytometry detection of mUCMSCs. The rates of mUCMSCs positive for CD34, CD90 and CD105 were 0.239%, 100%, and 99.9%, respectively. (**P**,**Q**) Isotype control. Osteogenic, adipogenic and chondrogenic staining of hUCMSCs (100× magnification). hUCMSCs were induced to differentiate into bone-like cells, adipose-like cells and chondrocytes. Alizarin red S staining, fatty oil red staining, and cartilage Alcian blue 8GX staining were used. (**U**) Control (normal cultured hUCMSCs). Osteogenic, adipogenic, and chondrogenic staining of mUCMSCs (100× magnification). (**V**) Osteogenic staining results. (**W**) Adipogenic staining results. (**X**) Chondrogenic staining results.

Both hUCMSCs and KM mUCMSCs were fibrous and adherent (Fig. 1A–D). The expression rates of the hUCMSC markers CD73, CD90 and CD105 were 97.5%, 98.2% and 97.5%, respectively, and the expression rates of CD34, HLA-DR and CD45 were 0.861%, 2.22% and 0.695%, respectively (Fig. 1E–L). The expression rates of CD105 and CD90 in KM mUCMSCs were 99.9% and 100%, respectively, while the CD34 expression rate was 0.239% (Fig. 1M–Q). Both types of UCMSCs could be induced to differentiate into adipocytes, bone-like cells, and cartilage-like cells (Fig. 1R–X).

### Model creation and evaluation

1. Acute IBD model: After 4 days of drinking 3% DSS solution, the mice developed blood in the stool. After 5 days, their body weight began to decrease, more blood appeared in the stool, and the mice became weak, drowsy. Gross anatomy: The whole colon, or sections of the colon, showed congestion, edema and ulceration. Histopathology and staining showed intestinal epithelial structure destruction, glandular disorders, a decrease in goblet cells, and a large amount of collagen fiber deposition in the whole layer of the intestinal wall (Fig. 2A–C). During the modeling period, the body weight, fecal morphology, blood in the stool, and survival rate were ascertained daily, and the DAI was calculated (Fig. 2D). The DAI of the model group compared to the control group has statistical significance (P = 0.012) (Fig. 2D).

**Figure 2:**
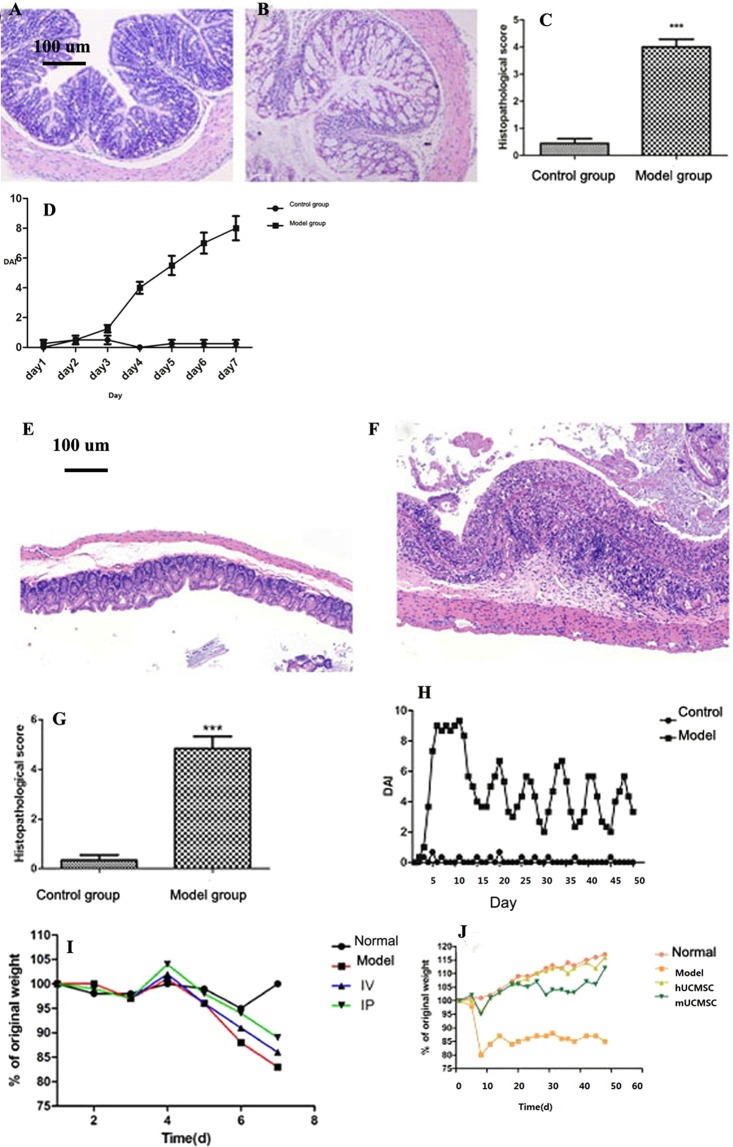
Pathological changes and analysis of colon tissue. (**A**) Normal control group; (**B**): model group (100× magnification); (**C**): ***P = 0.001; data are presented as the $\overline{{\rm{x}}}$  ± SD. In the model group, inflammatory cells infiltrated the muscularis and muscle layers of the mucosa and the glandular edema in the epithelium. DAI score. (**D**) During the modeling period, weight changes, fecal morphology, blood in the stool, and survival rate were measured every day, and the DAI was calculated. P = 0.012, the model group compared to the control group. Pathological changes in colon tissue. (**E**) Normal control group; (**F**): model group. In the model group, inflammatory cells infiltrated the whole layer of the intestinal wall, considerable cellulose exudation and cell proliferation in the intestinal wall caused swelling of the intestinal wall, and the intestinal lumen was narrow (100× magnification). In severe cases, the epithelial structure disappeared, and the organizational structure was unclear. Score. (**G**) Colon histopathology score. (**H**) Mouse DAI score. Changes in mouse body weight. (**I**) Changes in mouse body weight of the normal, model, IV, and IP groups. (**J**) Changes in mouse body weight in the normal, model, hUCMSC, and mUCMSC groups.
2. Chronic IBD model: The mice showed intermittent blood in the stool, and body weight remained low (approximately 85% of the initial weight). Inflammatory cells infiltrated the intestinal wall, the glandular structure almost disappeared, and the intestinal lumen was narrow (Fig. 2E,F) (P = 0.001). The mouse DAI was calculated during modeling (Fig. 2H). The change in body weight in each group was monitored daily from the first day of DSS administration. The average body weight changes in the acute and chronic IBD models are presented in Fig. 2I,J. During the observation period, the percentage weight loss in the IP, IV, and model groups was 11%, 14%, and 17%, respectively (Fig. 2I). The average body weight changes of each group in the acute IBD models has statistical significance (P = 0.035) (Fig. 2I). The average body weight changes of each group in the chronic IBD models has statistical significance (P = 0.025) (Fig. 2J). Further analysis of the mouse DAI showed that the IP injection improved diarrhea and blood in the stool, but these effects were not significant in the IV treatment group (Fig. 3A).

**Figure 3:**
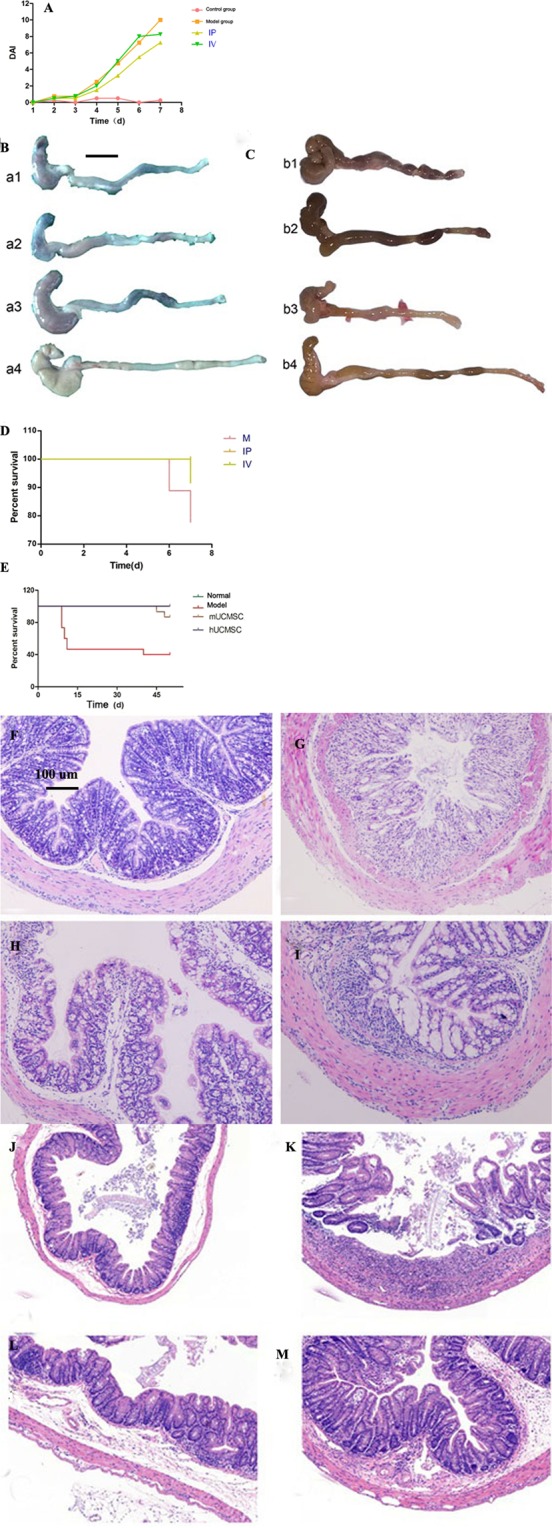
Mouse DAI. (**A**) IP, intraperitoneal injection group; IV, intravenous injection group. After IP injection, the mice showed improvements in diarrhea and blood in the stool, but the effects in the IV group were not obvious. Mouse colon anatomy. (**B**): a1, IV group; a2, IP group; a3, model group; a4, normal control group. (**C**): b1, b2, b3 and b4 represent the mUCMSC group, hUCMSC group, model group and normal control group, respectively. Mouse survival curve. (**D**): M, IP, and IV represent the model group, hUCMSC IP group, and hUCMSC IV group, respectively. In the acute IBD study, the survival rates of M, IV, and IP were 83.3%, 91.7%, and 100%, respectively (n = 12). (**E**) In the chronic IBD study, the survival rates of the normal control group (n = 9), model group, mUCMSC group and hUCMSC group were 100%, 40%, 86.7%, and 100%, respectively (n = 15). Values represent the average of each group. Colon histopathological changes (HE staining) (100× magnification). (**F**) Normal control group; (**G**): Model group; (**H**): IV group; (**I**): IP group. In the IP and IV treatment groups, leukocyte infiltration was observed in the colon, but the intestinal wall edema was mild, and the degree of glandular damage was significantly lower than that in the model group. In the model group, the intestinal epithelial structure was disordered, and edema, epithelial necrosis, cellulose exudation, inflammatory cell infiltration, and intestinal lumen stenosis were observed. (**J**) Normal control group; (**K**): model group; (**L**): hUCMSC group; (**M**): mUCMSC group. Observations of colon tissue in the model group in the chronic IBD study revealed missing gland-like structures, an unclear layer of the intestinal wall, exuded cellulose, and the dense distribution of inflammatory cells in various layers of the intestinal wall. (**K**) The colon tissue of mice treated with UCMSCs was clearly visible, and the extent and density of inflammatory cell infiltration were inferior to those in the model group (**L**,**M**).

### UCMSC efficacy

1. Efficacy of UCMSCs in the treatment of IBD: In the experiment on the use of hUCMSCs to treat acute IBD, the model group showed obvious ulceration of the colon, intestinal necrosis (Fig. 3B,a3). Congestion and edema were not appeared in the normal control group (Fig. 3B,a4). IP injection of UCMSCs caused mild hyperemia in the intestine (Fig. 3B,a2). In the IV group, intestinal edema and obvious intestinal congestion were observed (Fig. 3B,a1). In the experiment on chronic IBD, the large intestine shortened in the model group (Fig. 3C,b3), and the intestinal wall surface showed slight hyperemia in the cell treatment group (Fig. 3C,b1,b2).
2. Survival rates of each group: In the acute IBD study, the survival rates of the model, IV, and IP groups were 83.3%, 91.7%, and 100%, respectively (P = 0.034) (Fig. 3D). In the chronic IBD study, the survival rates of the normal control, model, mUCMSC and hUCMSC groups were 100%, 40%, 86.7%, and 100%, respectively (P = 0.038) (Fig. 3E).
3. Efficacy of human and KM mouse UCMSCs in the treatment of chronic IBD: Light microscopy images after colonic HE staining are shown in Fig. 3F–M. In the IP and IV UCMSC treatment groups, leukocyte infiltration was observed in the colon, but the intestinal wall edema was mild (Fig. 3H,I). The intestinal epithelial structure of the model group was disordered, and edema, epithelial necrosis, fiber exudation with inflammatory cell infiltration, and intestinal stenosis were all present (Fig. 3G). The histopathological scores are shown in Fig. 4A,B. In the colonic tissue from the chronic IBD model group, the glandular structure was absent, the intestinal wall tissue was unclear, exuded cellulose was observed, and inflammatory cells were densely distributed in various layers of the intestinal wall (Fig. 3J–M). The colonic tissue of mice treated with UCMSCs (i.e., hUCMSCs and mUCMSCs) was clearly visible, the extent and density of inflammatory cell infiltration were inferior to those in the model group (Fig. 3J–M), and the scores were also lower (Fig. 4A,B). The histopathological scores of the normal control, IP injection, IV treatment, and model groups were 0.5 ± 0.30, 5.9 ± 1.10, 8.7 ± 1.39, and 8.8 ± 1.33, respectively (p = 0.021) (Fig. 4A,B).

**Figure 4:**
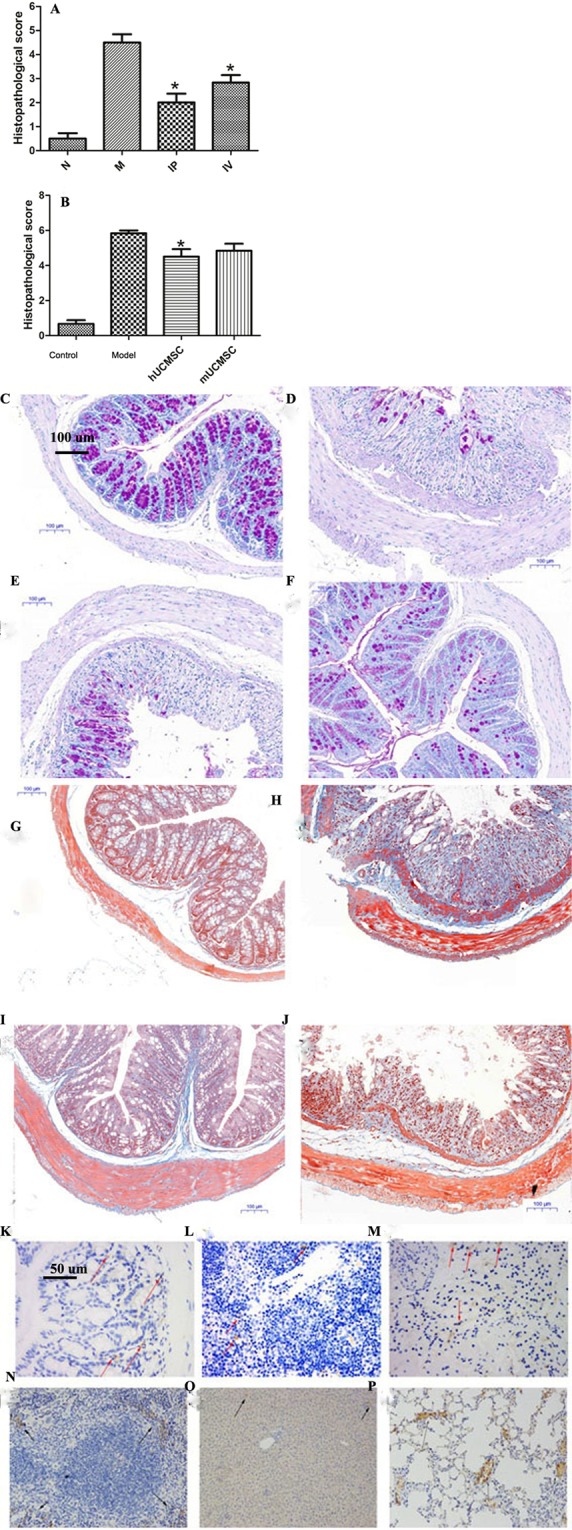
Statistical analysis of histopathological scores. (**A**) *P = 0.021 compared with the model group. Data are expressed as the $\overline{{\rm{x}}}$ ± SD. IP and IV indicate the UCMSC IP and IV injection groups, respectively. (**B**) *P = 0.032 compared to the model group. Data are expressed as the $\overline{{\rm{x}}}$ ± SD. Colon goblet cell staining (100× magnification). (**C**–**F**) Are the normal control group, model group, hUCMSC IV group and hUCMSC IP group, respectively. Glycogen granules in goblet cells are stained purple. After hUCMSC treatment of acute IBD model mice, PAS staining of the colon showed a diffuse reduction in or loss of goblet cells in the model group and more goblet cells in the UCMSC treatment group. Colon collagen deposition (100× magnification). In the acute IBD study, colon collagen staining showed that the collagen deposition area or range in the model group was significantly larger than that in the cell treatment group (**H**), indicating that the distal colonic hardening in mice in the model group is likely due to fibrosis. Collagen is stained dark blue. (**G**–**J**) are the normal control group, model group, hUCMSC IV group and hUCMSC IP group. UCMSC tracer. (**K**–**M**) Staining for UCMSCs in the mouse colon, spleen, and kidney. The brownish-yellow cell area is indicated by a red arrow (200× magnification). Trace staining of hUCMSCs. (**N**–**P**) Trace staining of hUCMSCs in spleen, liver and lung tissues. The brownish-yellow area (black arrow) indicates the presence of hUCMSCs (200× magnification). The staining of the spleen tissue was darker and more widely distributed, indicating the greater distribution of UCMSCs.
4. PAS staining of each group: In the acute IBD model, PAS staining showed a diffuse reduction in or loss of goblet cells in the model group and more goblet cells in the colon in the hUCMSC treatment group (Fig. 4C–F). The goblet cells in the colon of the hUCMSC and KM mUCMSC treatment groups were more evenly distributed at a higher density, and had less collagen fiber exudation than those in the model group (Fig. 4C–F).
5. Collagen staining of each group: After hUCMSC treatment of acute IBD, colon collagen staining showed a significantly larger area of collagen deposition in the model group than in the cell treatment group (Fig. 4G–J), indicating that the distal colonic sclerosis in the model group was probably due to fiber deposition.
6. UCMSC tracer: After UCMSCs were injected into the body, they distributed in the intestine, liver, spleen, lungs and kidneys, with the greatest distribution in the spleen (Figs. 4K–P and 5A,B).

**Figure 5:**
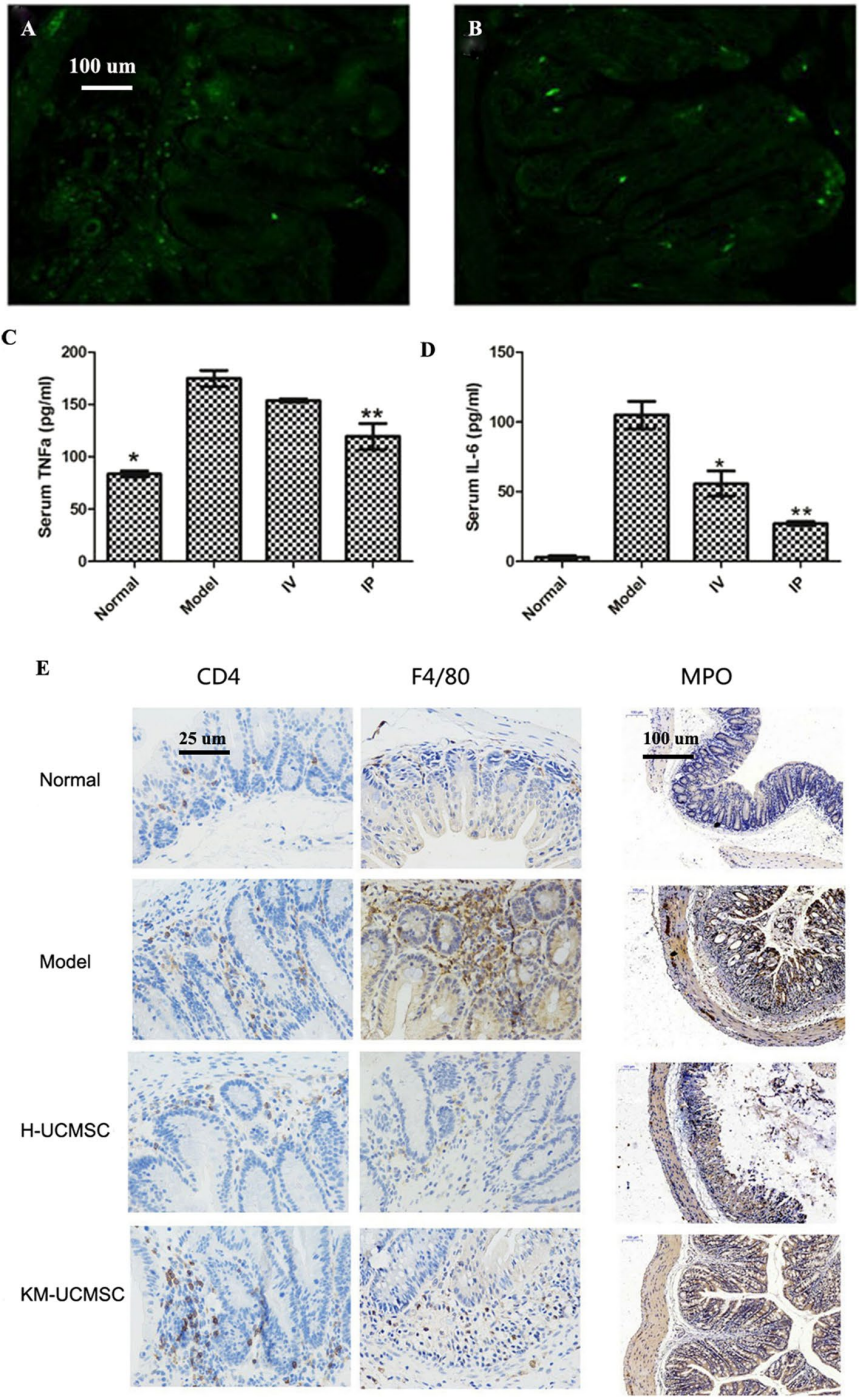
Distribution of GFP-labeled UCMSCs in various layers of the colon intestinal wall. (**A**,**B**) GFP-labeled MSCs were found in various layers of the intestinal wall. TNF-α and IL-6 concentrations in mouse serum. (**C**,**D**) Normal, model, IV, and IP indicate the normal control group, model group, hUCMSC IV group, and hUCMSC IP group. Compared with the model group, the IP and IV groups had significantly reduced TNF-α and IL-6 levels. Infiltration of CD4+ T cells, macrophages, and neutrophils in colon tissue (100× and 400× magnification). (**E**) CD4, F4/80, and MPO are markers of CD4+ T cells, macrophages, and neutrophils, respectively. The infiltration of CD4+ T cells, macrophages and neutrophils into colon tissue was lower in the hUCMSC and KM mUCMSC groups, with infiltration levels close to those in the normal control group, than in the model group, which showed diffuse infiltration.
7. UCMSC efficacy: The serum IL-6 concentrations in the IP UCMSC, IV UCMSC, and model groups were 27.17 ± 1.48, 71.11 ± 9.19, and 104.90 ± 9.78 pg/ml, respectively (p = 0.015) (Fig. 5D). The serum TNF-α concentrations in the IP UCMSC, IV UCMSC, and model groups were 119.50 ± 12.33, 153.67 ± 1.50, and 174.92 ± 7.72 pg/ml, respectively (p = 0.033) (Fig. 5C). There was less infiltration of CD4 + T cells, macrophages, and neutrophils into the colon of the human and KM mouse UCMSC groups, similar to the normal control group, while the model group showed diffuse infiltration (Fig. 5E). The qPCR results showed that claudin-1 and occludin were upregulated in the IP UCMSC group compared with the other three groups (p = 0.045) (Fig. 6A–C). LC3A/B expression was lower in the human and KM mouse UCMSC groups than in the other two groups. VEGF-A, VEGFR-1 and occludin protein expression was increased in the hUCMSC treatment group, with higher expression levels than those in the model group and the KM mUCMSC treatment group and similar expression levels to those in the normal control group (Fig. 6D).

**Figure 6:**
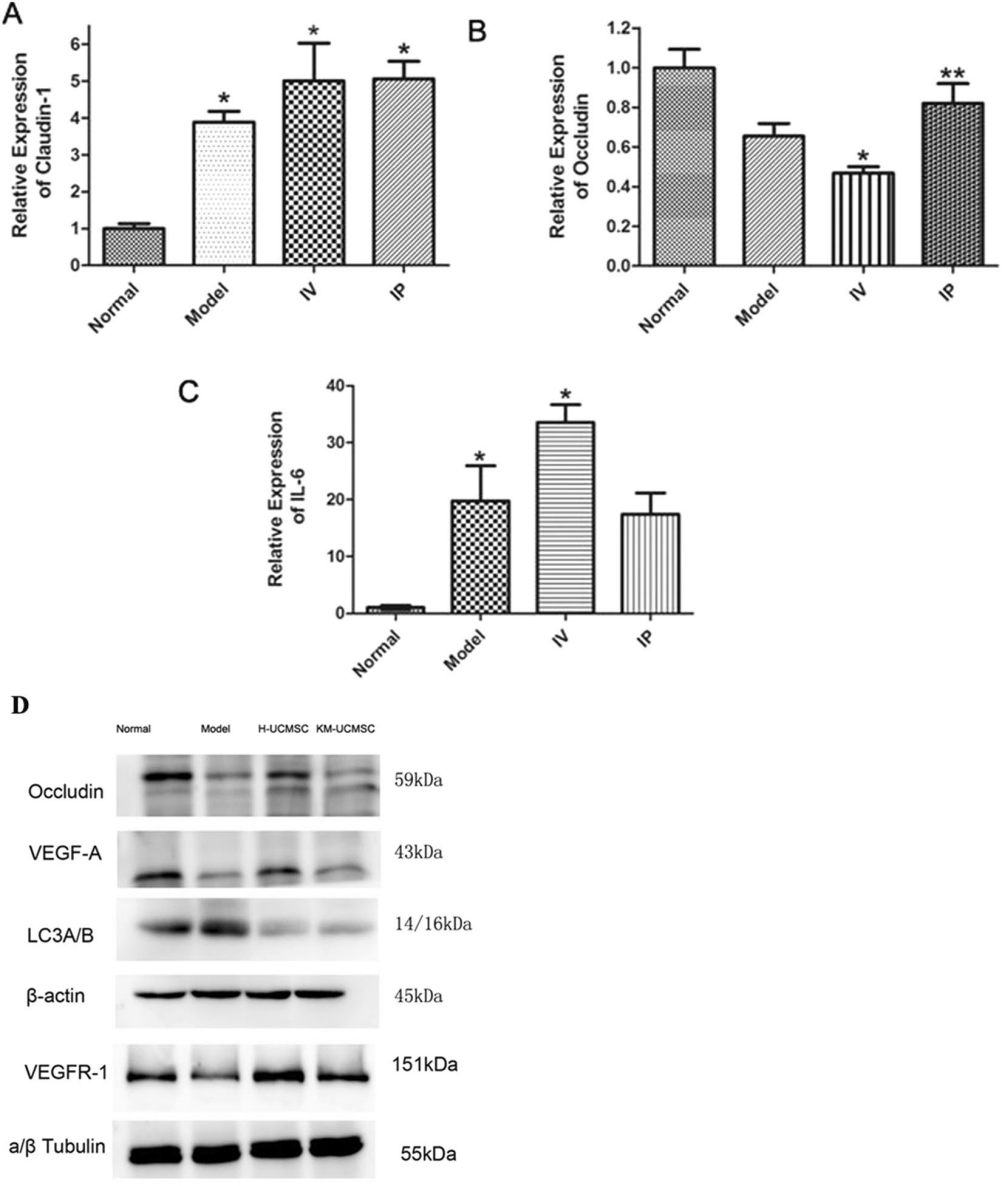
The relative gene expression of claudin-1, occludin and IL-6 in the mouse colon. (**A**–**C**) The claudin-1 expression level in the colon was significantly lower in the model group than in the cell treatment group (*P = 0.045). The occludin expression level was higher in the IP group than in the IV group (*P = 0.032). IL-6 expression in the colon was significantly increased in the IV group (*P = 0.023). Occludin, VEGF-A, LC3A/B, and VEGFR-1 protein expression in mouse colon tissue. (**D**) After the treatment period, the expression of occludin, VEGF-A and VEGFR-1 in colon tissue was higher in the hUCMSC group than in the model group and the KM mUCMSC group and close to that in the normal control group. LC3A/B expression was lower in the hUCMSC and KM mUCMSC groups than in the other two groups.

## Discussion

In this study, the efficacy of IV and IP MSC injections was compared. UCMSCs can promote the mRNA expression of claudin-1 and occludin, and the IP injection of MSCs can reduce the serum concentration of inflammatory factors such as IL-6 and TNF-α. Immunohistochemistry to localize MSCs revealed that MSC colonization was not limited to the injury site in the intestine upon IP or IV injection; these cells also localized to the spleen, lungs, kidneys and other organs. IP treatment was effective, and the efficacy of IV treatment was between that of IP treatment and model group. The DAI and pathological score were lower in the IP group than in the model and IV groups. Based on the above experimental results, we concluded that the IP injection of MSCs is not inferior to and even exceeds the traditional IV injection method. This study found that UCMSCs promoted the expression of the intestinal tight junction protein occludin, downregulated the protein expression of the autophagy marker LC3A/B in colon tissue, and upregulated VEGF-A and VEGFR-1 expression at the injured site.

There is no final conclusion as to which treatment route is safer or more effective. Some scholars believe that IV injections are superior to IP injections: the former approach can induce high levels of IFN-γ, thereby activating the immunosuppressive activity of MSCs and inducing T cell apoptosis at the injury site^22^. In contrast, Morgana *et al*. concluded that intraperitoneally injected MSCs home to the colonic lesion and reduce the inflammatory response, whereas intravenously injected MSCs are widely distributed in multiple organs outside of the colon^21^. A large number of experimental studies have fully confirmed the efficacy of IV or IP injections of MSCs^9,10,19^.

Regarding MSCs, we first think of their immunoregulatory function and tissue repair ability. In recent years, MSCs have emerged as candidates for the treatment of many diseases, for example, graft versus host disease (GVHD)^23^, CD^24^, type 1 diabetes^25^, allergic skin inflammation^26^, experimental autoimmune encephalomyelitis^27^, contusive spinal cord injury^28^, and acute pancreatitis^29^.

Accumulating data prove that MSCs must be stimulated to exert immunomodulatory activity. The most important stimulators are IFN-γ, TNF-α and IL-β^30–32^. In addition, signaling pathways mediated by Toll-like receptors (TLRs) can activate the immunosuppressive properties of MSCs^33^. Interestingly, MSCs can also act on host immune cells by inhibiting the proliferation of T cells and the maturation and function of dendritic cells (DCs); inducing the production of M2 macrophages, regulatory T cells and regulatory B cells; and regulating the biological behavior of NK cells.

In the cell-treated group, VEGF-A and VEGFR-1 protein expression levels were upregulated, indicating that MSCs contribute to capillary formation. Angiogenesis is a critical step in the tissue healing process^34^. Blood vessels transport nutrients to surrounding tissues and cells, and these nutrients facilitate cell proliferation and differentiation and other basic life activities.

There are many reports on the mechanism of MSCs in the treatment of diseases, including the following studies.

Li *et al*.^35^ reported oxidative stress–induced mitochondrial dysfunction can contribute to inflammation and remodeling in patients with chronic obstructive pulmonary disease (COPD). They found mesenchymal stem cells protect against lung damage in animal models of COPD.

Zhang *et al*.^36^ reported paracrine effect is the major mechanism that underlies mesenchymal stem cells (MSC)-based therapy. They found selective inhibition of Rap1 in BM-MSCs may be a novel strategy to enhance MSC-based therapeutic efficacy in myocardial infarction.

Liao *et al*.^37^ reported that transplantation of human induced pluripotent stem cell-derived mesenchymal stem cells (hiPSC-MSCs) is safe for the improvement of cardiac function in heart failure (HF). They found this is due to their immunomodulation that improves *in vivo* survival and enhanced angiogenesis via paracrine effects.

Chin *et al*.^38^ reported mesenchymal stromal cells (MSC) may improve cardiac function following myocardial infarction. We speculated MSC can differentiate into cardiomyocytes and endothelial cells while exerting additional paracrine effects.

Sun *et al*.^39^ demonstrate that, compared to adult MSCs, human iPSC-MSCs are insensitive to proinflammatory IFN-γ-induced HLA-II expression and iPSC-MSCs have a stronger immune privilege after transplantation. So we speculated it may attribute to a better therapeutic efficacy in allogeneic transplantation.

Zhang *et al*.^40^ reported at 4 weeks after cell transplantation, the nuclear casein kinase and cyclin-dependent kinase substrate 1 (NUCKS) (−/−)-BM-MSCs or wide type-BM-MSCs (WT-BM-MSCs) group significantly improved heart function and vessels density and reduced infarction size and apoptosis of cardiomyocytes. They demonstrated that depletion of NUCKS enhances the therapeutic efficacy of BM-MSCs for MI via regulating the secretion.

Yao *et al*.^41^ found that iPSC-MSC transplantation significantly reduced T helper 2 cytokines, and alleviated asthma inflammation in mice. They provided a therapeutic strategy for targeting asthma inflammation.

These articles all mention the mechanism by which MSCs treat disease, suggesting their broad application potential.

Based on the results of this study, we can infer the possible mechanism of UCMSC in the treatment of IBD: 1. UCMSCs promote the expression of the intestinal tight junction proteins occludin and claudin-1, reduce the permeability of the intestinal epithelium, and prevent intestinal endotoxins and other allergens from spreading to the blood and other organs, thus limiting the spread of inflammation. 2. UCMSCs downregulate the protein expression of the autophagy marker LC3A/B in the colon, thereby delaying the autophagic death of intestinal epithelial cells and helping maintain the mechanical barrier of the intestinal wall. 3. The UCMSC-induced upregulation of VEGF-A and VEGFR-1 expression in the damaged area contributes to microvascular regeneration, thereby improving local microcirculation in the intestinal wall and providing nutrients for the proliferation and renewal of intestinal wall cells.

## Conclusion

1. KM mUCMSCs were successfully obtained by tissue culture. mUCMSCs expressed high levels of CD90 and CD105 and low levels of CD34. hUCMSCs expressed high levels of CD73, CD90, and CD105 and low levels of CD34, CD45, and HLA-DR. Both KM mUCMSCs and hUCMSCs showed the potential to differentiate into bone, cartilage, and adipose cells.
2. The acute and chronic IBD mouse models were successfully established by continuous or intermittent administration of 3% DSS solution in drinking water.
3. IP and IV injections of UCMSCs for the treatment of acute IBD were effective, but the effect of IP injections was superior to that of IV injections.
4. Both hUCMSCs and KM mUCMSCs reduced enteritis and the mortality of the model IBD mice, suggesting that both sources of UCMSCs have therapeutic effects.
5. UCMSCs promoted the expression of the intestinal tight junction protein occludin, downregulated the protein expression of the autophagy marker LC3A/B in the colon, and upregulated the expression of VEGF-A and VEGFR-1 at the injured site.
6. After UCMSCs were injected into the body, they distributed into the intestines, liver, spleen, lungs and kidneys, with the greatest distribution in the spleen.

## Data Availability

All data generated or analyzed during this study are included in this published article.
